# Reaping the benefits of open science in scholarly communication

**DOI:** 10.1016/j.heliyon.2021.e08638

**Published:** 2021-12-18

**Authors:** Rosaria Ciriminna, Antonino Scurria, Sumalatha Gangadhar, Saikiran Chandha, Mario Pagliaro

**Affiliations:** a Istituto per lo Studio dei Materiali Nanostrutturati, CNR, via U. La Malfa 153, 90146, Palermo, Italy; b Typeset, 3260 Hillview Avenue, Palo Alto, CA, 94304, USA

**Keywords:** Open science, Open access, Preprint, Self-archiving, Scholarly publishing

## Abstract

Regardless of multiple efforts carried out across many countries to disseminate the ideas and the practice of open science, most scholars in the early 2020s do not self-archive their research articles and do not publish research papers in preprint form. Having received no education and training on open science, researchers are often puzzled on what to do, in practice, to start reaping the benefits of open science. This study offers a succinct *vademecum* on how to benefit from the open science approach to scholarly communication, no matter whether in natural or in humanistic and social sciences.

## 1 Introduction

The limits of conventional scholarly publishing as it actually developed in Europe in the late 1600s in the form of academic journals publishing scientific articles sent to academies in “sealed envelopes” were already known in the early 1800s to Évariste Galois. In 1831 the French mathematician explicitly called for a new scientific system in which “scientists will team up to study, instead of sending sealed envelopes to the academies, hastening to publish their slightest observations as long as they are new, adding: ‘I do not know the rest’” [1].

“Printing”, wrote Merton in 1973, provided the technology “for the emergence of that component of the ethos of science which has been described as ‘communism’: the norm which prescribes the open communication of findings to other scientists and correlatively proscribing secrecy” [2]. This “communication of findings”, however, has not been “open”, but rather limited to paying subscribers to the scientific journals in which said findings were published in the form of research papers.

In 1994, Harnad, a professor of cognitive science in Montreal, published in a mailing list a “subversive proposal” [3] asking researchers to make copies of all the papers they published in scholarly journals freely available on the Internet. “For centuries”, he wrote, “it was only out of reluctant necessity that authors of esoteric publications made the Faustian bargain to allow a price-tag to be erected as a barrier between their work and its (tiny) intended readership because that was the only way to make their work public in the era when paper publication (and its substantial real expenses) were the only way to do so. But there is another way today, and that is PUBLIC FTP: If every esoteric author in the world this very day established a globally accessible local ftp archive for every piece of esoteric writing he did from this day forward, the long-heralded transition from paper publication to purely electronic publication (of esoteric research) would follow suit almost immediately” [3].

Acronym of “file transfer protocol”, FTP is a computer protocol for file transfer used *inter alia* since the 1980s by computer scientists to share their research works in FTP archives, as well as by “high energy” particle physicists posting their works in the arXiv server since 1991.

In early 1993, the European Organization for Nuclear Research made freely available the source code of the “world wide web” invented by Berners Lee in 1991. The new “web” made even easier to share and access research articles on the Internet, when compared to FTP. Yet, little practical action followed Harnad's 1994 proposal for another two decades.

For example, out of nearly 1 million articles that could be self-archived in 2010, only 12% were actually self-archived by their authors [4].

Commenting on this outcome, in 2014 Harnad expressed his hope that “institutions and funders will now see to it that providing Green OA is effectively mandated before we lose yet another two decades of research access, uptake, usage, progress, productivity, applications, and impact needlessly” [5]. OA is the acronym of “open access”, a term adopted at a meeting of proponents of open access for scholarly journal literature attended by Harnad and other 15 delegates in Budapest in late 2001 [6]. “Green OA” indicates self-archiving of research articles on the author personal or institutional website following “green light” of the publisher (owner of the copyright) for making openly available on the web a research article published by a (usually paywalled) journal owned by the publisher.

Several excellent books [7], research articles [8, 9, 10, 11] and online presentations [12, 13] recount the history of open science and offer insight into its main concepts and objectives. In brief, open science aims to enhance the reproducibility of and accessibility to research findings by publicly exposing the scientific process by which they were obtained through four main practices [11]: open access, data sharing (open data), sharing procedures, methodologies and softwares (open source), and open peer-review (publishing the reviewer reports and the reviewer identities alongside the published article).

In addition to publications, several meetings on open science are regularly organized across the world which increasingly attract scholarly attention. For instance, the first edition of the OAI workshop series on innovations in scholarly communication held in Geneva in 2001 was attended by less than 50 people [14]. The 12^th^ edition held twenty years later had 1,400 registered delegates. The presentations given at these conferences usually made openly accessible on preprint platforms, and the video recordings published on the web are truly useful educational resources [15].

Regardless of these and related efforts to disseminate the ideas and the practice of open science, most world's scholars in the early 2020s do not yet publish their works in preprint form and do not self-archive their research articles, with entire research fields, like the basic science of chemistry [16], still dominated by the practice to publish research papers in paywalled journals. In this context, it is perhaps not surprising to learn that analyzing the 12,682 published articles dealing with “COVID-19” listed on PubMed (a biomedical scientific article database) as of July 1, 2020 and the “Altmetric” (a data science service tracking online mentions of published research) of all COVID-19 preprints found on arXiv, medRxiv, and bioRxiv, Besançon, Masuzzo and co-workers identified numerous cases pointing to “a very opaque peer-review process” with data often being not shared, poor adoption of preregistration reports leading to plentiful research duplication, and frequent misuse of preprints by journalists and news editors (with even incomplete OA to published articles that when granted “might have accelerated the search for solutions to the pandemic both in medical and socio-economic contexts”) [17].

Having received no education and training on open science, most scholars are often puzzled on what to do, in practice, to start reaping the benefits of open science. This study offers therefore a succinct *vademecum* on how to benefit from the open science approach to scholarly communication, no matter whether in natural or in humanistic and social sciences.

## 2 Self-archiving

Unknown to most scholars, publishers allow authors to self-archive their research articles in personal or institutional (repository) websites immediately or shortly after publication. Studying 1,150,827 articles published in 8,578 journals by the 100 largest publishers by article output in 2010 (42 commercial publishers, 52 professional associations or scholarly societies, and 6 university presses), Laakso found that nearly half (548,718) of all articles published by the aforementioned publishers in 2010 were permitted to upload *immediately* upon publication [4]. The share rose to 80.4% of all articles (924,725) after an embargo period of 12 months following online publication. Only 2.1% (24,188) of the articles were allowed to be posted online after a longer embargo. Laakso also unveiled that repository self-archiving was restricted by the 12-month embargo to a larger extent than author website self-archiving. The latter was rarely embargoed.

Five years later, the most common embargo period was 12 months for 62% of journals published by the top 107 publishers, with 20% allowing post-publication after 6 months [18]. Again, the analysis carried out for papers published in 2015 found that nearly 75% of publishers allowed authors to self-archive a version of their paper immediately on the personal author website [19].

Hence, the first practical tip to scholars willing to reap the benefits of open science is to open their own website and publish therein their own articles. Their peers indeed are less interested in articles deposited in a non-full-text mode, as it often happens with links to research articles found in institutional repositories [19].

The first benefit offered by self-archiving will be a rapid increase in citations. The OA citation advantage ensured by self-archiving varies amid disciplines, but it is generally significant. For example, articles in physics that have been made OA by their authors by self-archiving receive between 2.5 and 5.8 more citations than articles from the same journals that have not been made OA by their authors [20]. Furthermore, as shown by a regression analysis applied to 442,750 articles in 576 biomedical journals across 11 years, the citation advantage for green self-archived OA papers is independent of article age, journal impact factor, and number of co-authors [21].

So well known is (or should be) the citation advantage, that in 2016 the European branch of the Scholarly Publishing and Academic Resources Coalition, a Dutch foundation dubbed SPARC Europe “decided not to further update The Open Access Citation Advantage Service since the citation advantage evidence has now become far more common knowledge to our authors” [22].

Scholars, however, seem to be unaware of the benefits of self-archiving even in scientifically advanced countries. For example, the Canadian Institute of Health Research adopted an open access policy for its grant recipients in 2008 making mandatory the OA publication of research articles funded by the institution. Yet, out of 471 articles in 17 physical science research areas published between 2008 and 2015, 268 (57%) were not openly accessible. The remainder 43% share were openly accessible, but only 67 articles (14%) were self-archived at an institutional or subject repository [23]. Noting that this low uptake of the green open access route could not be ascribed to publishers’ archiving policies, since nearly all publishers allowed researchers to use green self-archiving, Zhang and Watson concluded that the results “speak to a need for education... given the low green open access deposit rate 9 years after the implementation of an open access policy” [23].

On the other hand, a 2014 study of 1,525 European highly cited scientists concluded that successful scientists systematically publicize their research by linking their online list of publications and their personal websites either directly to the self-archived articles or to subject repositories [24].

To publish and update their personal websites, scholars can either buy a domain name or use one of the numerous websites offering free hosting. Free hosting services offer website templates developed by professional designers, including themes for listing publications and conference presentations. For scholars willing to concisely display their team's work through an original format, numerous web page editing applications are freely available online to create an original and usable [25] website.

## 3 Immediate publishing of reproducible research

After having self-archived all published research, publishing new research in preprint form, namely posting online a research article immediately after completion of research in a specialized or cross-disciplinary preprint platform, is the second most important pillar of adopting the open science approach to scholarly communication.

**Figure 1:**
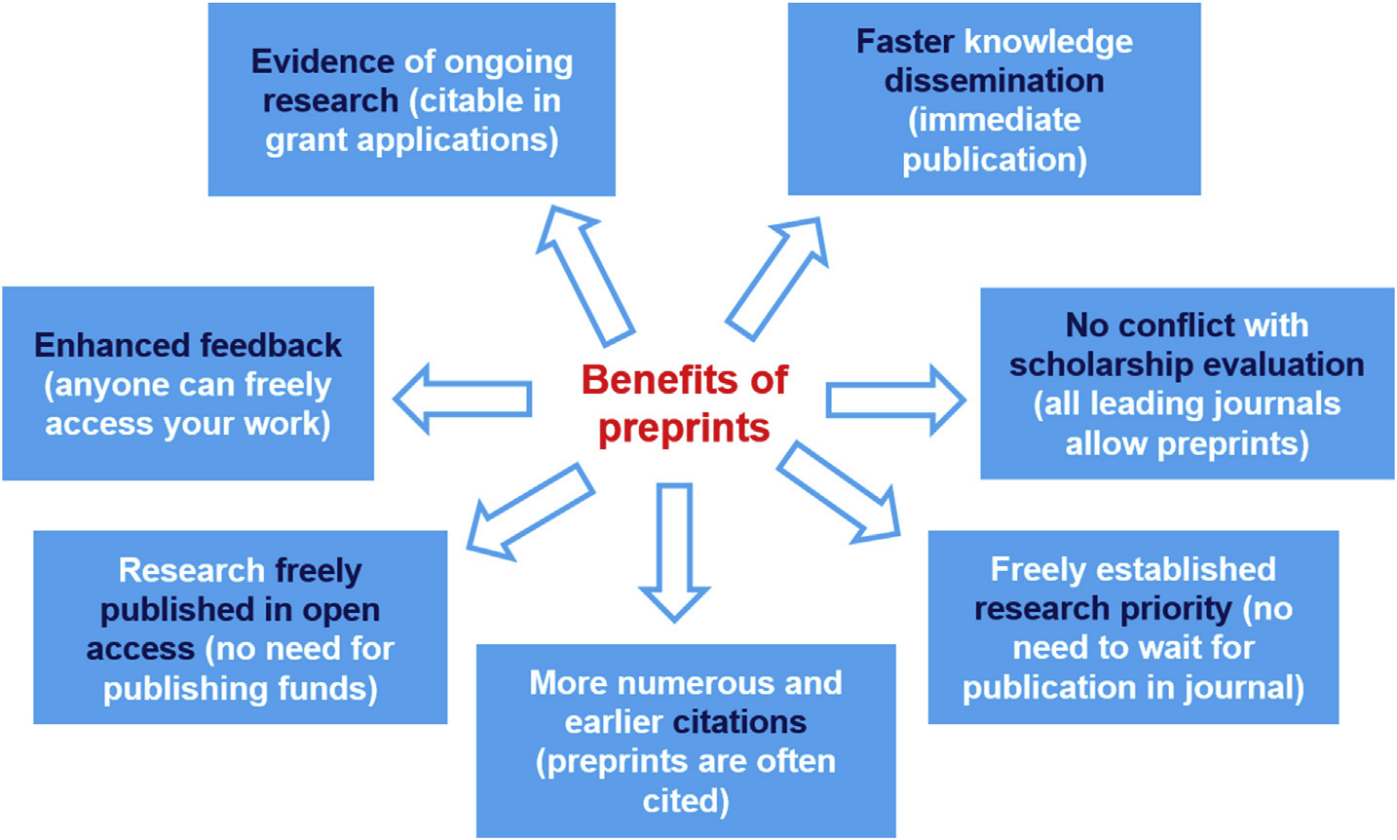
The main benefits of preprints.

The unique benefits of this scholarly communication means are now well established (Figure 1 and Table 1) [26].

**Table 1:** Features and benefits of preprints.

Feature	Benefit
Immediate publication	Faster dissemination of new knowledge
Receives a unique identifier	Earlier and frequent citations
Immediate and free publication	Credit and priority of the research unveiled
Freely accessible to anyone	Enhanced feedback to guide research improvement
Free open access publication	No need of publishing funds
Preprints accepted for peer review at all major scientific journals	No conflict with current scholarship evaluation practices
Preprints can be cited in grant applications	Evidence of ongoing research

Making research immediately and freely accessible to anyone, the preprint eliminates the prolonged delay due to the peer review process, thereby accelerating the dissemination of new knowledge. For example, the average delay between manuscript submission and journal publication ranges from 18 months in business/economics through 9 months in chemistry and biomedicine, 14 months in social science and arts/humanities, and 12 months in earth science [27].

In addition, preprints are readily and frequently cited. For instance, 69.1% of all preprints posted in arXiv and subsequently published as peer reviewed articles in *Physical Review D* between 1996 and 2012, received their first citation *before* they were published in the journal [28]. Besides physics, today preprints are widely read and cited also in the life sciences. For example, the 107,518 preprints posted at bioRxiv by the end of 2020 were cited 23,820 times [29].

Immediate publication allows authors to establish credit and priority for the new ideas, methods, and approaches disclosed by the preprint, providing “scoop protection” [30]. This is especially important for young researchers and for scientists devising significant advances in their research field.

Finally, no conflicts any longer exist between the early publication of research in preprint form and subsequent publication in peer reviewed journals. Nearly all major scientific journals not only accept preprint manuscripts for peer review, but their editors actually survey preprint research platforms inviting authors to submit selected preprints to their journal. Certain journals, such as *eLife*, have gone further and now accept for peer review *only* preprints [31].

Even chemistry journals, whose editors and publishers once fiercely opposed preprints, now actively encourage authors to submit their preprint [32]. The five world's five largest national chemical societies (United States of America, Great Britain, China, Germany, and Japan) jointly own a chemistry preprint server (ChemRxiv) which in its first four years has published 9,700 preprints from authors based in 100 countries [33].

The practice of open science through the immediate publication of new research findings in preprint form adds the crucial benefit of enhanced research reproducibility. Along with the preprint, indeed, scholars can freely publish also the underlying data, the protocols and software (code) used to collect and interpret the data, as well as links to the records for the dataset, deposited protocol, or to pre-analysis plans [34].

For example, the peer reviewed study on preprint credibility published by Nosek and co-workers in 2020 included the following statement: “The final survey included questions in four categories: engagement information, the importance of cues for credibility, credibility of service characteristics, and demographics (see https://osf.io/4qs68/for the full version of the questionnaire” [35]. The latter link directs readers to a preprint entitled “Credibility of Preprints Survey/Materials” [36] that in its turn includes all the methodology details on the survey of 3,759 researchers about their perceptions of the importance of different cues for assessing the credibility of preprints.

Similarly, a new way to make data and methods available and reproducible is the “executable paper”, namely a research article made available as an interactive digital document combining text, data, and code used for the analysis leading to the research conclusions [37].

## 4 Benefits in research evaluation

Emphasizing the relevance of the open science principles of reproducibility and transparency, Jon Tennant was used to highlight that “the opposite of open science is not closed science - it's bad science” [38]. The only reason for which the practice of open science continues to lag across many disciplines has been clearly identified in 2016 by McKiernan and co-authors: most researchers are “uncertain about how sharing their work will affect their careers” [10].

Yet scholars do not have to wait for the assessment criteria to change to start doing open science. This was recently shown by the quick policy reversal on a preprint ban in grant applications issued by the Australian Research Council (ARC) [39]. Called “plain ludicrous” [40], the ban for which more than 20 fellowship applications were deemed ineligible because they mentioned preprints and other non-peer-reviewed materials, was lifted on September 14, 2021. “This adjustment to ARC's policy position” read the announcement published online, “reflects contemporary trends and the emerging significance of preprint acceptance and use across multiple research disciplines as a mechanism to expedite research and facilitate open research” [41].

As suggested by a reviewer of this work, this single example shows that “if the community changes the practice, then the rules will follow”.

Regardless of numerous thoughtful pleas to institutions and funding agencies to diversify and improve research evaluation [42], researchers continue to be hired and promoted based on citation-based metrics. The researcher uncertainty “about how sharing their work will affect their careers” [10] can therefore be overcome by undertaking new, practically oriented education of undergraduate and doctoral students on the topic of open science, starting from scholarly communication in the digital era [43].

Amid the five main guidelines to shape the curriculum of such a course, Pagliaro has suggested to teach students the relevance of preprints and how to undertake regular publishing of research in preprint form [43].Attending a similar course, students for example will learn that, contrary to the aforementioned fears concerning their careers, the practice of open science leads to *enhanced* citations and citation-based metrics. Preprints indeed are frequently cited [29], and publishing a scientific study first in preprint form enhances also the number of citations of the so called “version of record” subsequently published in a peer-reviewed journal [44]. Similarly, OA peer reviewed papers not only receive more citations and online (social media) attention than non-OA papers, but OA articles are accessed and downloaded for a much *longer time* compared to non-OA papers [45].

Learning open science, doctoral students and researchers will become aware that rather than publishing preprints and research papers as portable document format (PDF) documents only, it is necessary to publish them also in a computer-readable markup language (HTML and its extensions) so as to make research data and meta-data easily retrieved by online search engines and databases, unlocking the accessibility of research findings. Being the equivalent of “a digital photograph of a piece of paper” [46], indeed, the non-actionable PDF file is not fit for sharing, finding and accessing research papers on the internet.

Accordingly, scholars may wish learn the use of one of today's numerous online web research authoring platforms (Overleaf, Typeset.io, TeXwork, etc.) through which research teams can work online without the need to exchange subsequent versions of the manuscript, or to follow journal guidelines and citation styles. It is enough to specify the desired format out of numerous journal formats, and the software will automatically format the document, and insert metadata to optimize its indexing by search engines [47].

## 5 Outlook and conclusions

Amid the current global search for new and effective ways to approach scientific research, higher education and service to society “in a context of digitalization and openness” [48], this study provides a succinct *vademecum* for the uptake of the main open science practices when dealing with scholarly communication.

Beyond scientific publishing, however, the practice of open science enhances work and outcomes also in the two additional areas of scholarly activity, namely education and service to society. How the practice of open science effectively enhances student education and learning and scholarly service to society in the context of new evaluation of scholarship in the open science age [49], will form the topic of a subsequent study.

## Declarations

### Author contribution statement

Rosaria Ciriminna, Antonino Scurria: Conceived and designed the experiments; Analyzed and interpreted the data.

Sumalatha Gangadhar, Saikiran Chandha and Mario Pagliaro: Analyzed and interpreted the data; Wrote the paper.

### Funding statement

This research did not receive any specific grant from funding agencies in the public, commercial, or not-for-profit sectors.

### Data availability statement

Data will be made available on request.

### Declaration of interests statement

The authors declare no conflict of interest.

### Additional information

No additional information is available for this paper.
